# The Prolyl Isomerase Pin1 Regulates mRNA Levels of Genes with Short Half-Lives by Targeting Specific RNA Binding Proteins

**DOI:** 10.1371/journal.pone.0085427

**Published:** 2014-01-09

**Authors:** Nithya Krishnan, Mark A. Titus, Roopa Thapar

**Affiliations:** 1 Hauptman-Woodward Medical Research Institute, SUNY at Buffalo, New York, United States of America; 2 Department of Structural Biology, SUNY at Buffalo, New York, United States of America; 3 Department of Biochemistry and Cell Biology, Rice University, Houston, Texas, United States of America; 4 Department of Genitourinary Medical Oncology, The University of Texas M.D. Anderson Cancer Center, Houston, Texas, United States of America; University of Cambridge, United Kingdom

## Abstract

The peptidyl-prolyl isomerase Pin1 is over-expressed in several cancer tissues is a potential prognostic marker in prostate cancer, and Pin1 ablation can suppress tumorigenesis in breast and prostate cancers. Pin1 can co-operate with activated ErbB2 or Ras to enhance tumorigenesis. It does so by regulating the activity of proteins that are essential for gene expression and cell proliferation. Several targets of Pin1 such as c-Myc, the Androgen Receptor, Estrogen Receptor-alpha, Cyclin D1, Cyclin E, p53, RAF kinase and NCOA3 are deregulated in cancer. At the posttranscriptional level, emerging evidence indicates that Pin1 also regulates mRNA decay of histone mRNAs, *GM-CSF*, *Pth*, and *TGFβ* mRNAs by interacting with the histone mRNA specific protein SLBP, and the ARE-binding proteins AUF1 and KSRP, respectively. To understand how Pin1 may affect mRNA abundance on a genome-wide scale in mammalian cells, we used RNAi along with DNA microarrays to identify genes whose abundance is significantly altered in response to a Pin1 knockdown. Functional scoring of differentially expressed genes showed that Pin1 gene targets control cell adhesion, leukocyte migration, the phosphatidylinositol signaling system and DNA replication. Several mRNAs whose abundance was significantly altered by Pin1 knockdown contained AU-rich element (ARE) sequences in their 3′ untranslated regions. We identified HuR and AUF1 as Pin1 interacting ARE-binding proteins *in vivo*. Pin1 was also found to stabilize all core histone mRNAs in this study, thereby validating our results from a previously published study. Statistical analysis suggests that Pin1 may target the decay of essential mRNAs that are inherently unstable and have short to medium half-lives. Thus, this study shows that an important biological role of Pin1 is to regulate mRNA abundance and stability by interacting with specific RNA-binding proteins that may play a role in cancer progression.

## Introduction

The peptidyl-prolyl cis/trans isomerase NIMA-interacting 1 (Pin1) regulates the activity of phosphorylated proteins that play critical roles in cell cycle progression, cell proliferation, signaling, and apoptosis [1], [2]. Pin1 catalyzes prolyl cis-trans isomerization about phosphorylated Ser/Thr-Pro bonds in its substrates, thereby promoting conformational changes that are essential for protein function. Pin1 is specific for phosphorylated Ser-Pro or Thr-Pro dipeptide motifs [3], and frequently acts with kinases and phosphatases in signaling pathways and to regulate transcription. Pin1 has been shown to catalyze substrate dephosphorylation [4], [5], [6], regulate protein stability and ubiquitination [7], [8], and influence cellular localization of its targets *in vivo* [6], [9].

Consistent with its wide-ranging effects on cellular metabolism, Pin1 is an important therapeutic target [10], [11], [12] in Alzheimer's disease [13], breast [14], [15], liver [16], prostate [17], [18], [19], lung [20], and colon cancers [2] as well as asthma [12], [21]. Drug companies such as Pfizer Global R&D [22], [23] and Vernalis Ltd [24], [25] as well as several academic laboratories [26], [27], [28], [29], [30] have targeted Pin1 for anti-cancer therapy. Pin1 is up regulated in several cancers, and its substrates include Cyclin D1, c-Jun, β-catenin, c-Myc, Raf kinase and p53. While over-expression of ErbB2 or Ras is sufficient to initiate breast cancer in female mice, cancer initiation is completely blocked in Pin1^−/−^ mice, an effect that appears to be mediated via negative regulation of cyclin D1 levels by Pin1 [31]. Pin1 also modulates the protein stability of estrogen receptor-alpha (ERα), an important biomarker for breast cancer, by blocking ERα protein ubiquitination [32] and transcription of the ERα mRNA [33]. Pin1 expression is positively correlated with tumor grade in human prostate cancer (PCa) and could be an independent prognostic marker for PCa progression [17]. Ayala et al [17] showed that patients with a Gleason score of 6 or 7 with high levels of Pin1 have a four-to-eight times higher level of PCa recurrence. Conversely, knockdown of Pin1 either in the human PCa cell lines LNCaP and PC3, or Pin1 ablation in PCa mouse models reduced cell proliferation and suppressed tumorigenic phenotypes. Pin1 has been shown to interact directly with phosphorylated Ser81 in the androgen receptor (AR) N-terminal domain, regulating AR's interaction with β-catenin and AR transcriptional activity [19], [34].

While the studies to date have focused on the role of Pin1 in regulating transcription and signaling pathways, the effect of Pin1 in modulating RNA-mediated gene expression is less well understood. Recent studies have shown that Pin1 plays an important role in the posttranscriptional control of mRNA stability via its interactions with a subset of RNA binding proteins [35], [36], [37]. Pin1 has been reported to associate with AU-rich binding factor 1 (AUF1) [38], [39] and K-homology splicing regulator protein (KSRP) [39], [40] to regulate the stability of *GM-CSF* [35], *TGF-β1* [41], [42], and parathyroid hormone (*PTH*) [36], [37], [43] mRNAs. These mRNAs have adenine and uridine-rich elements (ARE) in the 3′ untranslated regions (3′ UTRs) that form binding sites for AUF1 and KSRP. They regulate mRNA stability either by protecting the mRNA from exonucleases and hence increasing stability, or by recruiting components of the RNA degradation machinery such as the exosome, thereby promoting decay. Pin1 directly associates with AUF1 and KSRP and introduces conformational changes in these proteins, altering their interactions with mRNAs.

In addition to the ARE containing mRNAs, we have recently shown that histone mRNA decay is also regulated by Pin1 [6]. Histone mRNAs are a unique set of eukaryotic transcripts that are not polyadenylated and are synthesized primarily during S-phase [44]. These mRNAs have very short half-lives of approximately 40 min and 10 min during and at the end of S-phase, respectively [45], [46]. Histone mRNAs contain a 26 nt stem-loop sequence in their 3′ UTRs [44] which is the binding site for Stem-Loop Binding Protein (SLBP) [47] that regulates histone pre-mRNA processing [48], export [49], translation [50] and decay [51]. We recently showed that Pin1 directly associates with the phosphorylated SLBP-histone mRNA complex and dissociates SLBP from the histone stem-loop [6]. Down-regulation of Pin1 by siRNA or its chemical inhibition by the Pin1 inhibitor PiB stabilizes histone mRNAs and also increases the protein stability of SLBP [6].

The objectives of this study were two-fold: the first goal was to was to identify additional mRNAs that may be post-transcriptionally regulated by Pin1 thereby providing new information as to how Pin1 may regulate mRNA stability on a genome-wide scale. The second goal was to identify additional RNA binding proteins, in particular ARE binding proteins (ARE-BPs) that are possible Pin1 targets. To do this, we performed microarray analysis on untreated and Pin1 siRNA treated HeLa cells, and validated the microarray data by qRT-PCR of control and Pin1 siRNA treated HeLa cells, as well as HEK293 cells. The majority of mRNAs that showed altered abundance in response to a Pin1 knockdown were involved in Wnt and PI3K signaling pathways and cell adhesion. All core histone mRNAs were identified as Pin1 targets in this study, validating our previous report that Pin1 regulates histone mRNA turnover by binding SLBP. In addition, several ARE containing mRNAs showed altered mRNA levels in response to a Pin1 knockdown. We screened several ARE-BPs for interaction with Pin1 *in vivo* and identified ELAV-like protein 1 or Human antigen R (HuR) as a Pin1 substrate *in vivo*. HuR is an ARE-BP that binds cis-acting AU-rich elements and alters the stability of several mRNAs that play a role in cancer and inflammation. Not surprisingly, our screen identified *c-Fos, Il6, RGS2, NOTCH1*, and *DUSP1* as ARE containing mRNAs that are stabilized by Pin1. These mRNAs have previously been shown to be HuR substrates. We propose that SLBP, HuR and AUF1 are targeted by Pin1 *in vivo* to regulate the decay of the subset of mRNAs with short half-lives. Taken together, the data underscore the role of Pin1 in regulating the stability of several eukaryotic mRNAs, lending further support to the view that Pin1 inhibitors could be important for anti-cancer therapy.

## Materials and Methods

### a) Antibodies

Rabbit polyclonal anti-Pin1 and mouse monoclonal antihemagglutinin (anti-HA) antibodies were obtained from Santa Cruz. An in-house rabbit polyclonal anti-SLBP antibody made toward the C terminal 13 amino acids of human SLBP was used for the Western blots. Antibodies toward the following human proteins were a gift from Dr. Ann-Bin Shyu (University of Texas Health Science Center): goat polyclonal antibody towards the C-terminus of human FBP1, mouse monoclonal antibody towards KSRP, mouse monoclonal CUGBP1, goat polyclonal against a 20 amino acid C-terminal peptide of TIA1, goat polyclonal antibody towards 18 C-terminal amino acids of TIAR, rabbit polyclonal antibody against amino acids 166–285 of TTP, mouse monoclonal antibody towards HuR, and a rabbit polyclonal antibody towards BRF1.

### b) RNAi

Pin1 siRNA and control C2 RNAs were obtained from Dharmacon's ON-TARGETplus^TM^ siRNA collection (Catalog #003291). For the microarray studies and RT-PCR validation, the knockdown was performed in HeLa cells using Lipofectamine RNAiMax (Invitrogen) using a two-hit method [6]. The sequences of the four different siRNAs in the ON-TARGETplus^TM^ SMARTpool siRNA collection are 5′-CCACAUCACUAACGCCAGC-3′, 5′-GAAGAUCACCCGGACCAAG-3′, 5′-GAAGACGCCUCGUUUGCGC-3′, and 5′-GCUCAGGCCGAGUGUACUA-3′.

We have previously reported [6] that similar effects are observed on histone mRNAs irrespective of whether a single siRNA corresponding to the sequence 5′GCUCAGGCCGAGUGUACUAA-3′ or the pool of four different siRNAs is used, indicating the off-target effects are minimal. The second siRNA transfection was performed 48 hrs after the first hit and the cells were cultured for another 72 hrs before harvesting for microarray analysis or RT-PCR. To compare the effects of a single Pin1 knockdown with those of SLBP, AUF1, KSRP, and HuR single and double knockdowns, HeLa cells or HEK293T were treated with siRNAs towards the p37 subunit of AUF1, HuR, KSRP, SLBP, and Pin1, as indicated. RNAi was performed in using a 25 nM concentration of siRNA using the two hit method. siRNAs for AUF1, KSRP, HuR, and SLBP were obtained from the Dharmacon ON-TARGETplus^TM^ SMARTpool siRNA collection. Protein and RNA samples were harvested 72 hrs after the second hit for western blotting and RT-PCR, respectively.

### c) Microarray Analysis

HeLa cells were cultured in DMEM medium with 10% fetal bovine serum. Control and Pin1 siRNA treated samples were prepared in an identical fashion. For RNAi knockdown of microarray samples, 50 nM siRNA was used for each hit and the cells were harvested 72 hrs after the second hit. The efficiency of the siRNA knockdown was probed by western blotting and a 75–77% reduction in Pin1 mRNA levels was achieved as confirmed from the microarray analysis. The microarray data was repeated three times from independent siRNA treated samples. Total RNA from frozen cell pellets was prepared using the RNAeasy midi kits (Qiagen, Inc.) following manufacturer's instructions. After elution, RNA samples were concentrated by ethanol precipitation and resuspended in nuclease-free water. Before labeling, RNA samples were quantitated using a ND-1000 spectrophotometer (NanoDrop) and evaluated for degradation using a 2100 Bioanalyzer (Agilent Technologies). Samples are required to have a RIN >7, an OD 260:280 of 1.9–2.0, and an OD 260/230>1.8 for gene expression array analysis. Expression profiling was accomplished using the HumanRef-8 whole-genome gene expression array and direct hybridization assay (Illumina, Inc.). Initially, 500 ng total RNA was converted to cDNA, followed by *in vitro* transcription to generate biotin labeled cRNA using the Ambion Illumina TotalPrep RNA Amplification Kit (Ambion, Inc.) as per manufacturer's instructions. 750 ng of the labeled probes was then mixed with hybridization reagents and hybridized overnight at 58°C to the HumanRef-8 v3 BeadChips. Following washing and staining with Cy3-streptavidin conjugate, the BeadChips were imaged using the Illumina BeadArray Reader to measure fluorescence intensity at each probe. The intensity of the signal corresponded to the quantity of the respective mRNA in the original sample. BeadChip data files were analyzed with Illumina's GenomeStudio (v2010.1) gene expression module (v1.6.0) to report both un-normalized and quantile normalized, background corrected gene expression signal levels. Three experimental data sets were used and each dataset had three biological replicates for the control and Pin1 siRNA treated RNA. Good quality of microarray data was obtained in all cases. The data were filtered in Microsoft Excel to narrow down the gene set to genes that had a reliable signal and had a p value <0.02. Only those genes that showed at least a two-fold change between the Pin1 siRNA treated and control samples were retained in the final analysis. The microarray data reported here are publically available at the GEO database under the accession number GEO: GSE41868.

### d) Isolation of Total RNA for RT-PCR

HeLa cells or HEK293T were lysed in 1 mL of TRIzol Reagent (Invitrogen). 0.2 mL of chloroform was added per 1 mL of TRIzol Reagent, mixed vigorously and allowed to sit at room temperature for 2–3 minutes. The samples were centrifuged at 12000×g for 15 minutes at 4°C. RNA was precipitated from the upper aqueous phase using 1 mL of isopropanol. The samples were centrifuged at 12000×g for 10 minutes at 4°C. The RNA pellet was washed once with 2 mL of 75% ethanol, air-dried and dissolved in 25 microliters of diethyl pyrocarbonate (DEPC)-treated water. Total RNA obtained was purified using PureLink^TM^ Micro-to-Midi RNA Purification System (Invitrogen) as per manufacturer's instructions. All RNA samples used for RT-PCR had an OD 260:280 of 1.9–2.0 (NanoDrop).

### e) Quantitative RT-PCR

Real-time quantitative reverse transcription-PCR was performed either with a Biorad MyiQ™ Single-Color Real-time PCR System (at SUNY Buffalo) or an ABI 7500 Real-time PCR System (at UTMDACC). The cDNAs were synthesized from 1.5 ìg of total RNA using SuperScript III reverse transcriptase (200 U/ìl) (Invitrogen) with random hexamers. The gene-specific primers are summarized in Table S1 in File S1. The reaction components were added as follows: 5 µl forward primer (5 µM), 5 µl reverse primer (5 µM), 12.5 µl SYBR Green (Applied Biosystems), 1 µl cDNA, Taq polymerase, and water to 25 µl. The cycling profile for each run consisted of 50°C for 3 min, template denaturation at 95°C for 5 min, followed by 40 cycles of 95°C for 15 s, a primer annealing-elongation step at 60°C for 30 s using the default ramp rate. This was followed by a final step of 40°C for 1 min. All reactions were run in triplicate and the mean values were used in the analysis. The cycle threshold (C_t_) values were determined using the instrument software. The change in gene expression levels was determined by normalizing mRNA levels of the gene of interest to the mRNA level of the housekeeping gene glyceraldehyde-3-phosphatase dehydrogenase (GAPDH) using the comparative C_t_ method.

### f) Co-immunoprecipitation and Western Blot Analysis

HEK293T cells were cultured in DMEM medium with 10% fetal bovine serum. Cell extract was prepared from 1×10^7^ cells. Cells were lysed in RIPA buffer (50 mM Tris HCl pH 8.0, 150 mM NaCl, 1% NP-40, 0.5% sodium deoxycholate, 0.1% SDS, 0.2 mM sodium vanadate, 50 mM sodium fluoride, protease inhibitor cocktail) and spun at 13000× g for 10 min at 4°C. The lysate was pre-cleared and then incubated with an anti-Pin1 antibody or anti-HA (as a control), 25 μl Protein A/G Magnetic beads (Pierce) and 10 μg/mL RNaseA where indicated, overnight at 4°C. The next day, the beads were treated as per manufacturer's instructions using the Classic Magnetic IP/co-IP kit (Pierce). The bound proteins were resolved on a 15% SDS-PAGE gel and probed for bound proteins by western blotting with ECL.

## Results and Discussion

### a) Gene-expression profile of Pin1 knockdown cells

**Figure 1:**
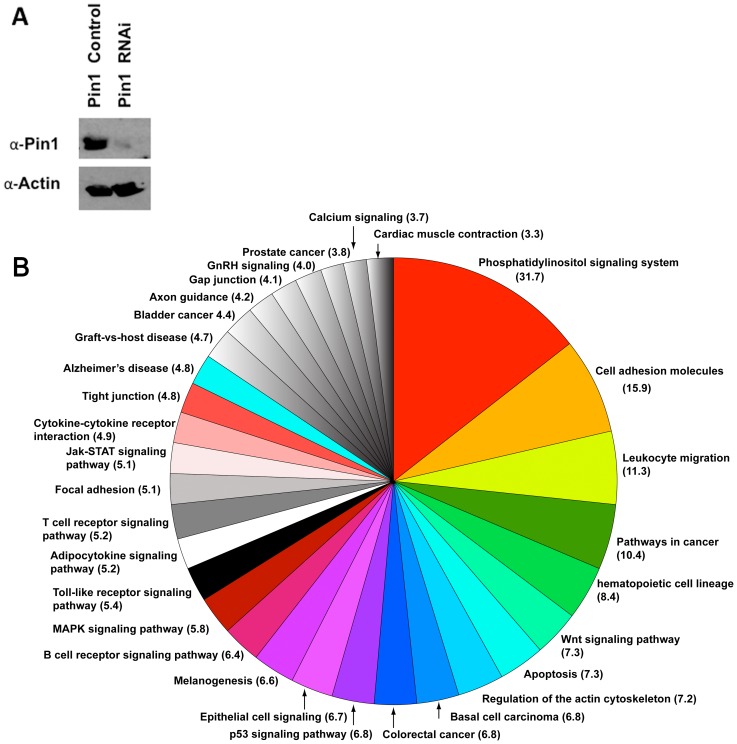
Pathway analysis of genes differentially expressed in Pin1 siRNA treated cells vs. controls. (A) Western blot showing the decrease in Pin1 protein levels in Pin1 siRNA treated cells used for microarray analysis. Actin is used as the loading control. (B) Functional clustering of genes based upon the major biological pathways targeted in Pin1 knockdown cells is shown. Pathway-Express software was used to identify pathways most affected by Pin1 silencing. The graph represent pathways ranked according to impact factor (in parentheses), a measure of the degree of pathway perturbation.

To identify mRNAs targeted by Pin1, we knocked down Pin1 in HeLa cells using RNA interference (RNAi) (Fig. 1A). We have previously characterized the effects of the Pin1 siRNAs used in this study on the cell cycle, histone mRNA abundance, and SLBP protein levels in both HeLa cells and HEK293 cells in detail [6]. As previously described [6], the S2 siRNAs used in this study were specific for Pin1. Western blotting and subsequent microarray analysis showed that at least ∼80% of the Pin1 protein was knocked down 72 hrs after the second siRNA hit using a 50 nM concentration of siRNA (Fig. 1A and [6]). RNA was extracted from these cells and hybridized to Illumina HumanRef-8 v3 BeadChips for microarray analysis. The microarray data was filtered to remove signal that was either close to background or was not statistically significant as ascertained by high p values. This analysis showed that Pin1 altered the mRNA abundance of 682 mRNAs that had p values <0.02 (90% with p values <0.01) and showed at least a two-fold change in the Pin1 knockdown cells relative to control cells. This indicates that only ∼3% of the transcriptome is under Pin1 regulation. Of the 682 mRNAs, 426 mRNAs were down-regulated and 256 mRNAs were up regulated. However whereas a 2–16 fold change was observed for those mRNAs that showed increased abundance (i.e. were up regulated), only a 2–4 fold difference was observed for most mRNAs that were down-regulated in response to a Pin1 knockdown. The 11 genes that showed at least a five-fold increase in their mRNA abundance were *CPA4, CDH5, CTGF, PSG4, EGR1, FOS, IL6, ETV5, M160, JUN*, and *CALB2*. Several of these genes have been implicated in cancer. Only two genes namely, *INHBE* and *STC1* were down-regulated by at least five-fold. The smaller change in mRNA abundance observed for down-regulated genes may be attributed to global changes in gene expression due to an altered cell cycle in response to a Pin1 knockdown. Gene ontology (GO) functional annotation using the Database for Annotation, Visualization, and Integrated Discovery (DAVID;http://david.abcc.ncifcrf.gov/) [52] showed that 52% of the genes were involved in signaling and apoptosis, 34% were involved in cellular metabolic processes, and 14% were involved in regulation of transcription, RNA processing, and nuclear transport. Analysis of cellular pathways using Pathway-Express [53] showed that for both up-regulated and down-regulated genes, cell adhesion, phosphatidylinositol signaling, leukocyte migration, Wnt signaling, pathways in cancer, apoptosis, and regulation of the actin cytoskeleton were highly perturbed by Pin1 gene silencing (Fig. 1B). Several pathways implicated in basal cell carcinoma, colorectal cancer, bladder cancer, prostate cancer, and Alzheimer's disease were impacted by Pin1 down-regulation. Signaling cascades under Pin1 control include the Wnt, MAPK, p53, Toll-like receptor, and JAK-STAT pathways.

**Figure 2:**
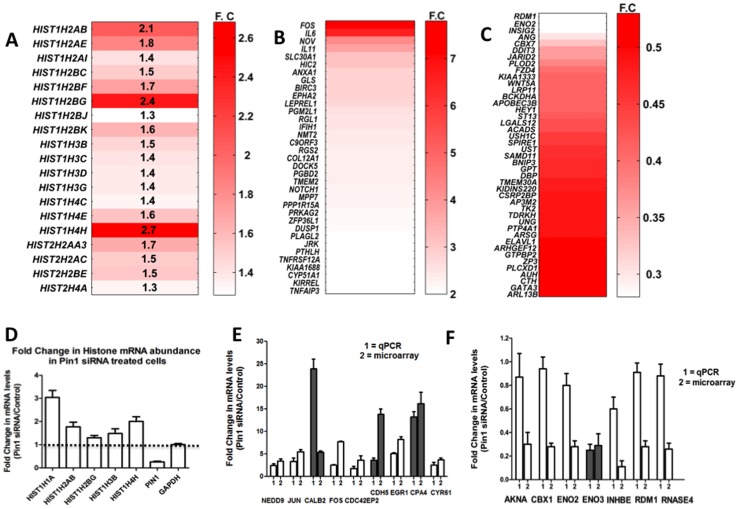
The mRNA abundance of histone mRNAs and ARE-containing mRNAs is altered in response to Pin1 silencing. Heat maps of histone mRNAs (A) and ARE-containing mRNAs (B and C) whose expression was significantly altered in Pin1 siRNA treated cells relative to control cells in the microarray data analysis. The heat maps depict the mean fold-change (F.C) for genes that had a p value <0.2. Panel B shows ARE containing genes that were up regulated in Pin1 siRNA treated cells, panel C shows ARE containing genes down-regulated in Pin1 siRNA treated cells. (D–F) The mRNA levels of a subset of genes that were significantly up or down-regulated in the microarray analysis were analyzed by RT-PCR as described in Methods. In (D), the fold change determined by RT-PCR for a subset of histone mRNAs is shown. Fig. D has been reproduced from (Fig. 7 panel A) Krishnan et al MCB (2012) 32:21, 4306–4322. In (E), data was validated by RT-PCR for a subset of genes that were up-regulated in Pin1 siRNA treated cells, whereas (F) shows data for genes that were down-regulated. Genes for which there was good agreement by both microarray and RT-PCR data are shown as filled bars.

The microarray expression data was validated by quantitative RT-PCR (qRT-PCR) for the histone genes (Fig. 2A and Fig. 2D) and genes that showed some of the largest changes in mRNA abundance by microarray analysis (Figures 2B, 2C, 2E, & 2F). These include *NEDD9, JUN, FOS, CALB2, CDC42EP2, CDH5, EGR, CPA4, CYR61, AKNA, CBX1, ENO2, ENO3, INHBE, RDM1*, and *RNASE4* (Fig. 2E & 2F). In general there was good agreement in the fold change in mRNA levels observed for up regulated genes between the two approaches (Fig. 2E). However, for genes down-regulated by Pin1 RNAi in the DNA microarray, the qRT-PCR data showed even smaller differences (Fig. 2F). We conclude that both microarray and qRT-PCR datasets show that Pin1 significantly increases the abundance of several mRNAs implicated in cancer. The microarray and qRT-PCR data is summarized in Fig. 1, Fig. 2 and the Tables S2, S3, S4 and S5 in File S1.

### b) The SLBP-Pin1 complex regulates decay of replication-dependent histone mRNAs

The replication-dependent histone mRNAs encode the core histones H2A, H2B, H3 and H4 that make up the nucleosome as well as a fifth histone H1 that is the linker histone essential for formation of higher-order structure in chromatin. In humans, a multigene family consisting of at least 65 histone genes encodes these five classes of histone proteins [54]. There are 55 histone genes that comprise the *HIST1* cluster on human chromosome 6 (6p21–p22), 6 histone genes form the *HIST2* cluster on chromosome 1 (at 1q21), and 3 histone genes form the *HIST3* cluster on chromosome 1 (at 1q42). These replication dependent histone mRNAs are cell cycle regulated and are not polyadenylated [44]. Instead of a polyA tail, they have a conserved stem-loop in the 3′ untranslated region (3′ UTR) that binds Stem-Loop Binding Protein (SLBP) [47]. The histone mRNA stem-loop/SLBP complex coordinates the expression of histone genes during S-phase of the cell cycle so as to synchronize it with DNA synthesis. The replication-dependent histone mRNAs are regulated primarily at the level of mRNA processing and mRNA decay. The mature histone mRNA increases 35-fold during the G_1_/S-phase transition and decreases back to G_1_ levels in late-S phase. These are short-lived mRNAs and have half-lives of 40 min during S-phase and only 10 min at the end of S-phase, respectively. In addition to the replication-dependent histones, there are also some replacement variant histone genes such as H2a.X, H2a.Z, and H3.3 that are polyadenylated and are not cell-cycle regulated.

Our microarray analysis showed that 15/55 genes belonging to the *HIST1* cluster and 4/6 genes from the *HIST2* cluster were stabilized in response to a Pin1 RNAi knockdown. We did not detect any genes from the *HIST3* histone cluster or variant histone genes by microarray analysis. The microarray data was validated for the core histones H2A, H2B, H3, and H4 by qRT-PCR (reported here as Fig. 2D and Table S7 in File S1. We also previously reported this data in Krishnan et al MCB (2012) 32:21, 4306-4322 as Fig. 7, panel A). In addition, we also determined that the linker histone *HIST1H1A* was stabilized 3-fold by qRT-PCR, although it showed poor signal in the microarray dataset. All core replication-dependent histone mRNAs (H2a, H2b, H3, and H4) showed an increase in mRNA abundance in Pin1 RNAi treated cells (Fig. 2A, Fig. 2D and Table S7 in File S1). The observed increase in abundance was between 1.4–2.7 fold by microarray analysis (Fig. 2 and Table S7 in File S1) and was validated by qRT-PCR to be between 1.3–3.0 fold (Fig. 2D). Since histone mRNAs are expressed only during S-phase, and Pin1 RNAi knockdown cells show fewer S-phase cells [6], this represents approximately a 1.7–4.0 fold change in histone mRNA abundance overall. This apparently modest change in steady-state mRNA levels is biologically significant since an excess of histone protein leads to misregulation of DNA packaging and proper chromatin assembly eventually leading to cell death. Consistent with this result, previous studies report two or three-fold changes in histone mRNA steady state levels due to alterations in regulated decay pathways that affect histone mRNA turnover [51].

**Figure 3:**
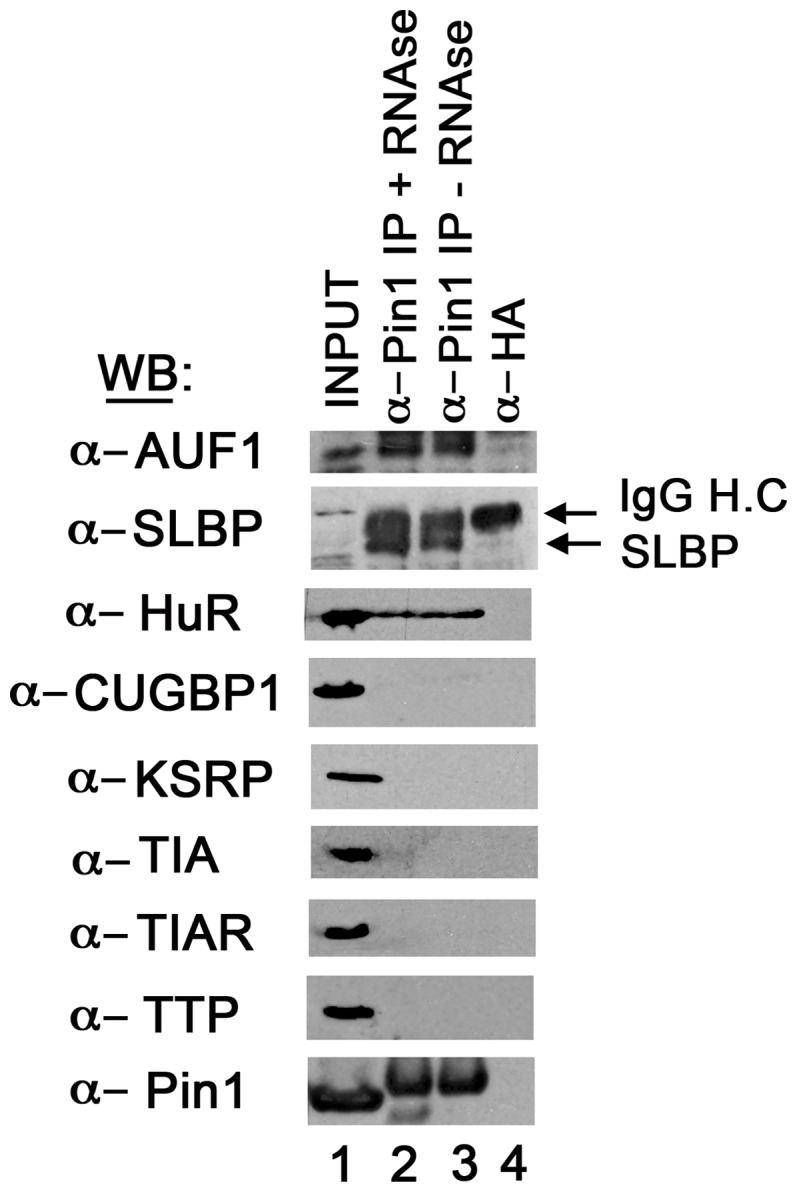
Pin1 interacts with a subset of phosphorylated RNA binding proteins. Cell lysates from 293T cells were immunoprecipitated with either an anti-Pin1 or anti-hemagglutinin (HA)-specific antibody. The immunoprecipitates were resolved on a 15% SDS-PAGE and analyzed by western blotting for several ARE-BPs, Pin1, and SLBP. Five percent of the input sample was analyzed in lane 1, proteins bound to the anti-Pin1 antibody in the presence (lane 2) or absence of RNAseA (lane 3), and proteins bound to the anti-HA antibody in the absence of RNAseA in lane 4.

The increase in mRNA abundance of 20 histone mRNAs in this study validates our recently published study [6] that showed that Pin1 directly binds SLBP (also shown in Fig. 3 of this paper) and dissociates the histone mRNA/SLBP complex at the end of S-phase, regulating histone mRNA decay and SLBP protein stability. In the Krishnan et al [6] report, we determined that the effects of Pin1 siRNA on histone mRNA abundance could be mimicked by chemical inhibition of Pin1 by PiB, and that the increased abundance of histone mRNA was due to regulation of histone mRNA decay. Histone mRNA decay rates decrease significantly for all five histones in the presence of PiB and the rates are also sensitive to hydroxy urea (HU), which triggers rapid decay of histone messages. The increased abundance of histone mRNA could be rescued by over-expression of Pin1 in siRNA treated cells, indicating that the effects of Pin1 on histone mRNA levels were specific and not due to off-target effects.

**Figure 4:**
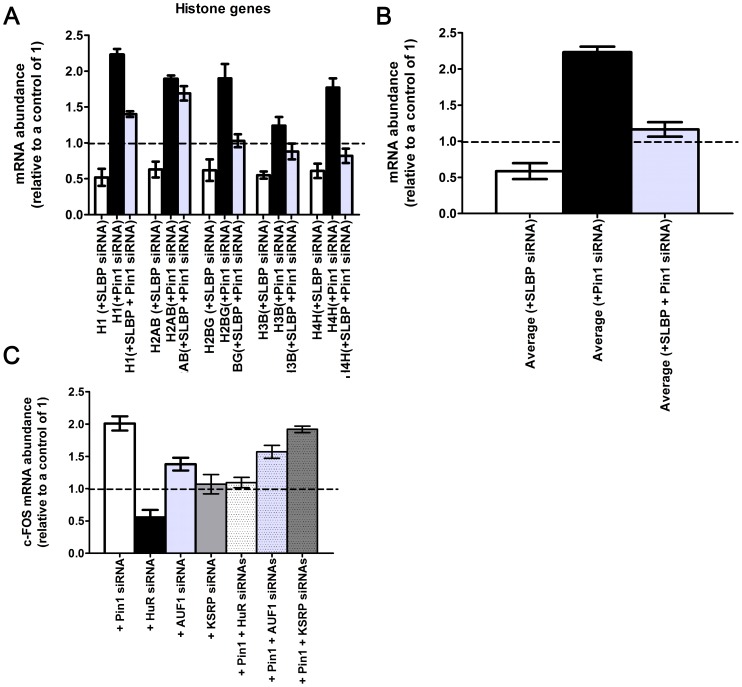
Pin1 acts in concert with specific RNA binding proteins to regulate the mRNA abundance of histone mRNAs and the *c-FOS* mRNA. (A) qRT-PCR analysis of histone mRNA expression in response to siRNA knockdown of Pin1, SLBP, and a double Pin1/SLBP RNAi knockdown in HEK293T cells is shown for a subset of histone mRNAs. The average change in histone mRNA levels for all five histone genes probed is shown in (B). In (C), qRT-PCR analysis of the c-FOS mRNA in response to siRNA knockdown of Pin1, HuR, AUF1, KSRP, and the double Pin1/ARE-BP RNAi knockdown in HeLa cells is shown.

To further determine whether there was any synergy between Pin1 and SLBP in regulating histone mRNA abundance, we knocked down these two genes either independently or together using RNAi (Fig. 4A and Fig. 4B). When HEK293T cells are treated with a pool of four different siRNAs specific for SLBP, the histone mRNA abundance decreases to 52–63% of control as measured by qRT-PCR. This effect is similar to that previously reported [49] and is attributed to a decrease in histone pre-mRNA processing in SLBP depleted cells. However, in HEK293T cells treated with siRNAs towards both Pin1 and SLBP, the histone mRNA abundance increased to between 100–200% of control levels, indicating that the stabilization afforded by Pin1 is enough to compensate at least partially the effects of the SLBP siRNA. Taken together with previous studies, the data suggests that Pin1 acts with SLBP to regulate histone mRNA levels *in vivo*.

### c) Pin1 targets several ARE-containing mRNAs via its interaction with AUF1 and HuR

In addition to histone mRNAs, the mRNA abundance of several AU-rich element (ARE) containing genes was altered in Pin1 siRNA treated cells (Fig. 2, Tables S4 & S5 in File S1). Approximately 9114 genes i.e. ∼50% of human protein coding mRNAs are computationally predicted to have ARE sequences in their 3′ UTR in the Adenylate Uridylate (AU)-Rich Element Database (ARED, http://brp.kfshrc.edu.sa/ARED/). Several of these mRNAs encode oncogenes, cytokines, and cell cycle regulators that play important roles in cancer progression. These mRNAs are known to undergo rapid mRNA deadenylation and degradation that is trigged by the associated ARE-BPs. Only 35 ARE-containing mRNAs (representing only 0.4% of the predicted ARE containing mRNAs) showed increased abundance and 43 ARE-containing mRNAs (representing 0.5% of the total) showed at least a two-fold change in abundance in response to a Pin1 knockdown. As discussed earlier, the fold change in mRNA levels was greater for those mRNAs that showed increased abundance. With the exception of c-JUN (a class III ARE containing mRNA), all of the ARE containing transcripts we identified belong to either the class I or class II family which are characterized by the presence of multiple copies of the pentanucleotide sequence AUUUA, that is absent in the class III family. Therefore the effects of Pin1 appear to be specific for only a subset of ARE containing mRNAs. Some ARE-containing mRNAs identified in our study that have well-established roles in oncogenesis include *c-FOS, IL-6, IL-11, NOV, HIC2, ANXA1, EPHA2, DOCK5, NOTCH1, RGS2, RDM1, ENO2, CBX7, FZD4,* and *WNT5A* (Tables S4 and S5 in File S1). The change in mRNA abundance for several of these mRNAs was validated by qRT-PCR (Fig. 2E, 2F and Table S6 in File S1). The expression of IL-6 and IL-11 mRNAs was found to increase six and four-fold, respectively, in Pin1 siRNA treated samples. The data is consistent with previous reports [55] that showed increased levels of inflammatory cytokines such as IL-6 in Pin1 −/− mice (compared to wild type mice) in response to induction of inflammation by lipopolysaccharides. Interestingly, Pin1 siRNA knockdown also down-regulated the ARE-BP HuR (ELAV1) mRNA by two-fold. HuR is a member of the ELAV (embryonic lethal abnormal vision) family of RNA binding proteins that stabilize several important mRNAs, protecting them from decay. As discussed below, Pin1 also directly binds the HuR protein, regulating its activity to control the decay of the *c-FOS* mRNA. Therefore our results indicate that Pin1 regulates the activity of the HuR protein and also down-regulates its mRNA. Consistent with this, our screen identified several mRNAs whose decay is regulated by HuR, such as *c-Fos, Il6, RGS2, NOTCH1*, and *DUSP1*.

To identify ARE-BPs that directly associated with Pin1, we immunoprecipitated Pin1 from 293T cells (Fig. 3) and screened the immunoprecipitates for the presence of AUF1, HuR, TTP, TIA1, TIAR, CUGBP, FBP, and KSRP. All of these ARE-BPs are phosphoproteins and have phosphorylated Ser-Pro/Thr-Pro sequences that are required for Pin1 association. However only AUF1 and HuR were found to associate with Pin1 in our study. This association was RNA-independent as addition of RNAseA did not disrupt the complex. AUF1 has already been shown to bind Pin1 to regulate the activity of the GM-CSF [35], [56] and Pth [36] mRNAs. However HuR has not been previously shown to bind Pin1. Interestingly, AUF1 and HuR frequently bind to the same ARE-containing mRNAs (class I and class II), with AUF1 being a decay promoting factor and HuR inhibiting mRNA decay. Although KSRP has been reported to bind Pin1 [36], we did not observe this association in HEK293T cells under the conditions of our experiment.

To determine whether AUF1, HuR, and KSRP could synergize with Pin1 to regulate mRNA stability, we knocked down these genes by RNAi either separately or in conjunction with Pin1 in HeLa cells (Fig. 4C). Since the mechanism of ARE-mediated decay is well understood for the *c-FOS* mRNA, we sought to determine whether the *c-FOS* mRNA levels were altered in response to the different siRNA treatments. siRNA knockdown of Pin1 increased *c-FOS* mRNA levels by two-fold whereas siRNA knockdown of HuR resulted in a two-fold decrease in *c-FOS* mRNA levels, consistent with previous studies [57]. This decrease is likely due to an increase in mRNA decay as the role of HuR in regulating the *c-FOS* gene is well established [58]. siRNA knockdown of the p37 subunit of AUF1 showed a smaller (∼40%) but significant increase in stability of the *c-FOS* mRNA, while siRNA knockdown of KSRP had no effect on mRNA levels. A Pin1/KSRP double knockdown showed the same two-fold increase in *c-FOS* mRNA levels consistent with no appreciable role of KSRP in regulating the *c-FOS* mRNA stability. Intriguingly, for both Pin1/HuR and Pin1/AUF1 double siRNA knockdowns, the *c-FOS* mRNA levels increased but were less than Pin1 siRNA alone. The mechanisms by which Pin1 may act on HuR and AUF1 are presently unclear. Pin1 could act by dissociating AUF1 from the *c-FOS* mRNA thereby stabilizing the message as has been reported for the GM-CSF mRNA [35]. On the other hand, HuR is phosphorylated at two Ser-Pro sites (Ser221 and Ser202) both of which regulate HuR cellular localization. Phosphorylation at Ser221 by PKC has been implicated in translocation of HuR to the cytoplasm [59], [60] whereas phosphorylation at Ser202 by Cdk1/Cdc2 is important for nuclear retention of HuR during G2/M phase of the cell cycle [61], [62]. Pin1 could regulate the cellular localization of HuR by facilitating phosphorylation or dephosphorylation of HuR. Future studies will test how Pin1 acts in concert with AUF1 and HuR to exert its effects on the *c-FOS* mRNA.

### d) Is there a correlation between Pin1 levels and mRNA half-lives?

**Figure 5:**
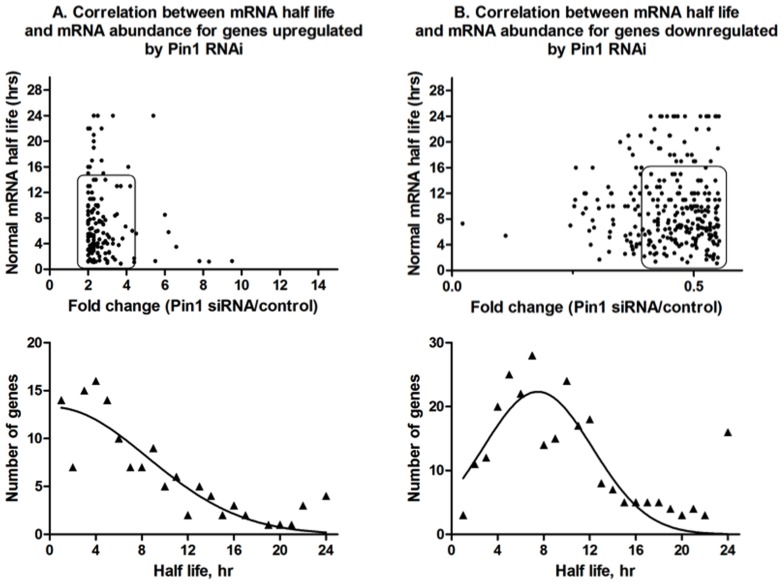
Correlation between mRNA half-lives of the target genes identified and change in mRNA abundance in the Pin1 siRNA knockdown. *(Top)* In (A), the fold change in mRNA abundance for genes whose mRNA levels increase at least two fold is plotted against their half-life. In (B), the fold change in mRNA abundance for genes whose mRNA levels decrease at least two fold is plotted against their half-life. *(Bottom)* Frequency distribution of target genes that are either stabilized (left) or destabilized (right) in Pin1 siRNA treated cells as a function of mRNA half-life. Genes that show the largest fold stabilization in a Pin1 siRNA knockdown have half-lives <4 hr.

A remarkable finding of our study is that the mRNAs whose abundance is significantly altered (p<0.02) by Pin1 silencing include 20 histone mRNAs and 78 ARE-containing mRNAs. These mRNAs have short half-lives. To determine whether these mRNAs were particularly sensitive to Pin1 levels on a global scale, mRNA half-life was analyzed against a change in mRNA abundance in the Pin1 RNAi treated cells relative to control (Fig. 5). The median half-life of all mRNAs sensitive to Pin1 was found to be 5.6 hrs. However, mRNAs that showed the most significant increase in abundance in the presence of the Pin1 siRNA had half-lives shorter than 2 hr. Only 11% (n = 24) of genes with half-lives >12 hr showed a statistically significant change in mRNA abundance and this change was small (only around two-fold), suggesting it may represent a more non-specific global effect of treatment with Pin1 siRNA. These effects become more apparent when the frequency distribution of genes is plotted as a function of half-life (Fig. 5A, bottom panel; R^2^ = 0.8). Short-lived transcripts (half life <6 hr) comprise at least 50% of the total pool of mRNAs that are stabilized in response to Pin1. We conclude that Pin1 may act to specifically modulate the stability of mRNAs whose levels need to be tightly regulated. In the case of histone, GM-CSF, and TGFβ mRNAs, the effects of Pin1 on mRNA abundance have been shown to be due to decreased decay of the mRNA [6]. However, a similar analysis of mRNAs that were down-regulated in response to Pin1 siRNA (Fig. 5B) showed a distribution that centered around a two-fold change in mRNA abundance suggesting these effects are more likely due to global, cell-cycle dependent changes rather than any specific effect. There was a poor correlation (R^2^ = 0.6) between change in mRNA abundance and half-life for genes down-regulated by Pin1. The median half-life of mRNAs that were down-regulated in response to Pin1 siRNA was between 6.2–8.8 hr. We conclude that Pin1 plays an important role in regulating the stability of a subset of eukaryotic mRNAs via its interaction with the associated RNA binding proteins.

## Supporting Information

File S1 **Supporting Tables.** Table S1 The primer sequences used for RT-PCR. Table S2 List of mRNAs whose abundance increased by Pin1 siRNA knockdown. Table S3 List of mRNAs whose abundance decreased by Pin1 siRNA knockdown. Table S4 ARE containing mRNAs stabilized by Pin1 siRNA knockdown. Table S5 ARE containing mRNAs destabilized by Pin1 siRNA knockdown. Table S6 Change in mRNA abundance for several genes measured by qRT-PCR. Table S7 Change in mRNA abundance for histone mRNAs measured by microarray and qRT-PCR.
(XLS)
